# Elements of Chemistry, in the Order of the Lectures Given in Yale College

**Published:** 1831

**Authors:** 


ART. IV.—*Elements of Chemistry, in the order of the Lectures given in Yale College, by* BENJAMIN SILLIMAN, Professor of Chemistry, Pharmacy, Mineralogy, and Geology.—2 vol. oct., p. 1262.

Within a very few months, several valuable works on Chemistry, have been issued from the British and American press.—The system before us, however, has the merit of being the most extensive publication, of American origin, with which we are acquainted. The well earned reputation of the author, had secured for his work, long before its appearance, a highly favorable presentiment. He had long enjoyed the reputation of originality, and of great practical skill; and his fame, in the important business of inventing and simplifying the utensils of the laboratory, was almost unexampled. He had, moreover, furnished the scientific community, with many valuable papers on philosophical subjects: and the way was thus prepared for a most flattering reception of a full treatise on his favourite science.

The first volume treats of the *imponderable agents*, in which are included, light, caloric, attraction, &c. Of *ponderable bodies*, as oxygen, nitrogen, hydrogen, alkalies, earths, simple inflammable and acidifiable bodies, (not metallic,) and their combinations with the preceding bodies.

Professor Silliman states his reasons for departing from the practice of other writers, in noticing *seriatim*, the simple substances, in the following words:—"I have not thought it best to describe the simple substances in uninterrupted succession.—Such a method does not appear to me, to present advantages sufficient to compensate for the inconvenience of plunging, at once, into the most complex parts of the science, which must be done, if we would draw the elementary bodies from their combinations, and present them, in the beginning, in a connected view."

I do not, however, perceive the force of this objection, to what is obviously the most rational arrangement. That it is possible for a lecturer or writer, to treat the various subjects of Chemistry, in such a progressive manner, as that the law of passing from the known to the unknown, shall never be violated, no one will presume to affirm. But as it is obviously correct, to teach a child the alphabet, before the spelling book is placed in his hands, so it seems to me, to be equally proper, to discuss the nature of simple bodies, before an effort is made to investigate the character of compounds, whose constituents have not been, as yet, under examination.

We object also, to the method pursued, in relation to the class of bodies, called *salts*; and the mistake now referred to, obtains in the second volume, to a greater extent, than in the first. Here, however, the author is not alone, for the fault is one of very general occurrence. Thus, instead of going regularly through the history of the alkalies and earths, and noticing all their saline compounds, in one chapter, we find ammonia, potash, and soda, treated of, in one place, and their salts scattered through the volume, as if there was no natural relation between them.

We prefer greatly a plan, very different from that of our author. Thus, after examining oxygen and nitrogen, treat of the atmosphere; notice hydrogen, and then examine the properties of water; then the compounds of oxygen and nitrogen, embracing the several acids, thence resulting. Then carbon, and its compounds, with oxygen, nitrogen, and hydrogen, and so on of all the simple substances. In this way, we become acquainted with all the acids, and are prepared to examine the metals.— Let the alkaline and earthy metals be first examined, in connection with their oxydes and salts; thus, in treating of potassium, notice its combinations with oxygen, and then the various salts resulting from the union of potash, with the several acids; and thus let the whole catalogue of metals be investigated. I am sure, from some little experience in teaching, that this method is better calculated to advance the student, than any other with which I am acquainted.

There is so much force in the *Rules of philosophising*, page 173, vol. 1st, that we venture to insert the remarks, without abbreviation or comment.

1. God is the first cause of every thing.

(*a*.) *All our observations, experiments, and reasonings, make us acquainted only with second causes.*

(*b*.) *The proximate cause of an effect, is the only immediate antecedent to the event, or which is principally operative in producing it.*

(*c*.) *To every proximate cause, there may be another proximate cause, and to that cause another; but the series will end at last in the power of the Creator, in immediate agency; and this will still be the fact if we discover ever so many proximate causes, constituting a series of chain, apparently endless.*

(*d*.) *When we have classified similar phenomena, and have discovered their modus operandi, we say that we have found out the law that governs them;* but still this harmony of facts and operations, we must trace to the same source.

(*e*.) *Natural science is to be studied by observing facts, and making experiments, and then drawing conclusions;* this is the inductive or Baconian method of reasoning and is the foundation of a legitimate theory. An experiment is nothing but the exhibition of a fact.

(*f*.) *Hypotheses may be introduced in the absence of true theory founded on induction;* but they can be admitted only provisionally, until something better can be done.*

(*g*.) We will add from Sir Isaac Newton, that "*we are to admit no more causes of natural things, than such as are both true and sufficient to explain their appearances.*"

(*h*.) "*Therefore, to the same natural effects we must, as far as possible, assign the same causes.*"†

(*i*.) *The range of human reason is the whole extent of second causes.*

(*j*.) *The final reason of a particular law is sometimes discovered by us*, and always magnifies the author. The unvarying proportion of oxygen gas in the atmosphere, and the means by which it is probably sustained; the exception in the expansion of water between 32° and 40°; the phosphorescence of marine animals, and of fish generally,

*See Lord Bacon's Novum Organum, and De Augment. Scientiarum.

†Principia, Vol. II, Ed. 1803.

in the ocean, and the circulation of fluids and of aeriform bodies in currents, to equalize temperature, are striking instances among multitudes that might be adduced.*

(*k.*) *The moral effect of physical study upon every mind which has been correctly disciplined, is altogether happy, and augments the vigor of every proper feeling.*—It is not, however, to be denied, that an opposite effect is sometimes produced upon certain minds; but this is the fault of the individua , and not of the study. Even moral study sometimes produces the same effect.

(*l.*) *The greatest mental power and the longest life, joined with the greatest industry, can enable man to compass only a small part of universal knowledge.*—Of this, the wisest and the greatest men are the most sensible. Newton was not more distinguished for his vast powers and acquirements, than for his singular modesty. The important suggestions at the end of his optics are in the form of queries. The whole amount of the knowledge of such a man, compared with all that a savage knows, is indeed great; but, compared with universal knowledge, it is an evanescent point."

Professor Silliman is correct in affirming, "that much may be done, in chemical investigation, by cheap and simple means." He informs us, "that Dr. Priestley's implements were gun barrels, tubes, flasks, vials, and corks; and yet few men have ever made more discoveries." We recollect, distinctly, the paucity of articles employed by Professor Cooper, who, as an instructive lecturer, has never been excelled in this country. Indeed, it has occurred to us, in more than one instance, that the splendour of apparatus, was designed to supply the place of an easy and felicitous method of instruction. It is certainly an advantage to an apt teacher, to have ample implements, so that he may be able to select such as may be most suitable. But it is equally certain, that the glare and lustre of costly utensils, cannot compensate for defective, half digested, and crude instruction.

Much exception was taken to the volume now under consideration, because of its servile imitation of Professor Hare's "Compendium," by some individuals, who find it easier to object, than to project. There is to be sure, an extensive system of copying apparent, especially in relation to plates of new in-

*Paley's Natural Theology.

struments, and of novel modes of manipulation. But, there is also evinced the most ingenuous spirit of acknowledgment, and in no instance is the author's name withheld. And let me inquire, what writer in Chemistry has not adopted the very course complained of? Is it practicable for every new treatise-maker, to give new diagrams, or to present original views? If our forthcoming works were thus restricted, they would be *only* forthcoming, and would never see the light. But if I mistake not, there are several of the implements, noticed in the volume before us, and for which credit is given to Dr. Hare, which ought to be regarded in the light of a joint stock, between that gentleman and our author. Certainly, the large *pneumatic cistern*, and to some extent, the *compound blowpipe*, are of this character. As a most barefaced larceny has been attempted, in relation to the last named instrument, and as the coadjutors in this unprincipled business, seem indisposed to do justice, though often reproved, it is with peculiar pleasure, we quote, at length, the testimony of a competent witness in the case. Professor Silliman thus speaks in p. 224, vol. i;

"Dr. Hare *first* ascertained, that oxygen and hydrogen gases can be made to burn together in this manner; (viz. by the compound blowpipe,) that the heat thus evolved, surpasses that produced by any other mode of combustion, and that it is scarcely exceeded even by that of voltaic electricity. Being an independent, original witness to the early use of this fine instrument [1802] by its inventor, and having been in the habit of using it frequently, for several years before Dr. Clarke's experiments were published, as well as ever since; I embrace this opportunity to say, that no other name than that of Dr. Hare can be, in my view, rightfully associated with the invention of the compound blow-pipe."

But the friends of Dr. Clarke are resolved to crown him with, at least, half the glory of this discovery, and exultingly affirm, that he is entitled to the credit of inventing a more powerful machine, by forcing the mixed gasses into a *common* vessel.—This, to be sure, is a little different from Dr. Hare's instrument, but the only advantage that we can perceive in the difference is, that Dr. Clarke has had ingenuity enough to make a blowpipe, which pretty certainly insures a *blow-up* to the machine, if not to the operator. Explosions have frequently occurred in its use; but we have yet to learn, when and where the compound blow-pipe of Dr. Hare, has met with this accident.

On the subject of acidity, Professor Silliman has the following remarks, vol. 1, page 306—"Some chemists are *now* inclining to the opinion, that no one principle can be regarded as being endowed with the peculiar prerogatives of being an acidifier; but that acidity may, and often does arise from a balanced or conjoined effect of several principles." He then refers to an ingenious discussion of this point, by Mr. Murray, in the last edition of his "Elements," (6th Edit. vol. 2, art. acids.) But if a suggestion be worth any thing, (and who does not know that discoveries have had, for their base, mere suggestions,) then it is proper that the maxim, *honor to whom honor is due*, should be observed. On this ground, the reviewer puts in his claim, and quotes from the "Memoirs of the Columbian Chemical Society," page 187, the following remarks, viz: "There is nothing, in my opinion, radically wrong, in the whole antiphlogistic system, except it be the admission of a principle of acidity. I believe acidification to be one of the simplest and most intelligible processes in nature or art. What is an acid but an assemblage of several kinds of matter, so arranged, as to constitute an homogeneous compound? And is it asked, to what I attribute the difference in acids? I answer, it is dependent on the variation in quantity of the different matters entering their composition. It has been conjectured, and that with some probability, that all matter was originally resolvable into three great constituents, oxygen, metallic base, and electricity. Hence, if this be a true conjecture, we may attribute the immense variety in acids, to the infinite modifications of these original substances. We see something like this, on a small scale, in the different compounds of nitrogen and oxygen, and in some other cases.

Oxygen has been deemed so essential to the formation of an acid, that some acid bodies have been considered as anomalies, because oxygen could not be detected in them. Now the view I have taken of the subject will obviate this difficulty entirely. For although I believe, that oxygen does probably exist in every body, possessed of acid properties, yet I do not consider that opinion at all impaired, by one or two apparent exceptions.—Prussic acid is said to be a compound of carbon, hydrogen and nitrogen, each of which is equally indispensable, to constitute it an acid. Now, one of these is as much entitled to the appellation of *acidifying principle* as the other, if such a term were at all philosophical." The above remarks were part of an *Analysis of Professor Coxe's Essay on Acidification and Combustion*, published in the year 1813, and agreeing precisely with the reviewer's statement, in his *Inaugural Dissertation on Acidification and Combustion*, dated March, 1812. In the N. Y. Medical Repository, A. D. 1813, the same writer published a paper, "*to determine, wherein consists the quality or principle of any body*," in which the same points are defended. Thus, on page 125, it is observed; "those who attempt to find out the seat of a quality, (or principle) would do as well to undertake the analysis of a quality itself. It is, as we have already remarked, something produced by variety in the union and arrangement of particles." The above quotations are of older date, by several years, than the remarks of Professor Murray, already adverted to.

In noticing the alkaline metals, Professor Silliman follows the usual course, of ascribing all the praise of decomposition to Sir Humphrey Davy. I have been in the habit of regarding this subject in a light somewhat different, for the last eighteen years, and am not disposed now to change my views. When supposed revolutions are effected in science, there is a prevailing disposition, to neglect authorities, which, from the very fact of such revolutions, are classed among the *obsolete*. This has been remarkably the case, in regard to the truly philosophical chemistry of Lavoisier, which, as an examplification of genuine tact in the science, has never, in my opinion, been equalled in any age. In that excellent treatise are to be found, some important statements, on the metallic character of the earth and alkalies. I am well aware, that a part of the book, called the *Elements of Chemistry*, was not written by Lavoisier, but the whole is so entirely devoted to the antiphlogistic chemistry, as to give to the production, a oneness of character which has justified the appellation so uniformly given to it.

In a note, at the foot of page 213, the translator says, "there are some experiments related in the Transactions of the Turin Academy, which give reason for suspecting, that soda is a modification of magnesia: this latter substance, according to the experiments detailed by Baron Born, seems to be a *metallic* oxyd. From analogy, we may presume that *potash* is also a metallic substance, in some hitherto unknown state of combination. We shall thus exclude all the alkalies from the class of simple elementary substances." In page 217, Lavoisier writes thus. "It is extremely probable that barytes, which we have just arranged with the earths, is an oxyde; for in many experiments, it exhibits properties really approaching to those of metallic bodies. It is even possible that all the substances we call earths, may be only metallic oxydes, irreducible by any hitherto known process." A section is added by the translator, *on the metallic nature of the earths*, in which, after detailing the processes by which the metallic bases of *lime*, *barytes*, and *magnesia*, were repeatedly obtained, he uses the following language: "should they eventually be confirmed by rigorous examination, a new light will be thrown on several of the most difficult parts of chemistry, by these discoveries, which have already been in a great measure predicted by the conjecture of M. Lavoisier, who supposes that those substances, which have long been considered as primitive earths, are only metallic oxydes, combined with oxygen, and that their reduction has hitherto been prevented by the attraction which subsists between them and oxygen, being stronger than that between oxygen and carbon." To this account, Baron Born adds, "that he expects soon to learn, that the siliceous and argillaceous earths are likewise metallic oxydes; and that, in this case, the whole class of earths and stones will disappear from the mineral kingdom."

There is reason to believe, that Dr. Woodhouse, formerly of the University of Pennsylvania, was the first person who supposed that potash had an inflammable base, as the result of experiment. He was operating with pearlash and soot, exposed to a very high degree of heat, in a covered crucible, and perceived, that water thrown on the mixture, when quite cold, caused it to take fire. He repeated the experiment with charcoal, and the same result followed. He had never seen potassium, nor heard of it; but who can now entertain a doubt, but that he actually produced that substance, and that the combustion which occurred under his notice, was the effect so frequently seen in later times, of potassium on water?*

All the facts above detailed, were matters of record, in books accessible by the English philosophers, at least thirty years ago, and of course, prior to the date of Sir Humphrey Davy's alleged discoveries. I say *alleged* discoveries, for I go on the justly established principle, of *rendering to Cæsar the things that are Cæsar's*. I admit cheerfully, that Sir Humphrey did very much to unfold the true character of the alkalies and earths; and am disposed to award him the meed of praise on all suitable occasions; but I hesitate not to affirm, that it was ungenerous and unworthy of the character of a philosopher, to treat with silent contempt, all that had been done in the premises, by men devoted to science, at a period anterior to the time, when he announced to the world the result of his labours. Nor does it better become the admirers of that philosopher, to persist in a similar neglect of the actual achievments of Lavoisier, and the advocates of his system, and to detract from their merits, by throwing around Sir Humphrey Davy's fame, a light, which owes a part of its brilliancy, at least, to the previous labors of equally devoted men. Again and again have I searched in new chemical works, to find a recognition of the facts I have endeavoured to bring to public view, but in vain, and I have been forced to the conclusion, that their authors had never read the *Elements of Lavoisier* with that attention which they obviously merit, or that so long a period had elapsed since the perusal, that the contents were wholly effaced from their memories. The best advice that I can give them in this matter is, to take up the book again, and to read it attentively and thoroughly, and then

* Vide Nicholson's Journal, page 240.

to determine whether the basis of chemical science, as it came from Lavoisier's hands, is radically changed by all that Sir Humphrey Davy has done.

I am fully aware of the operation of the chloridic theory, and of the supposed influence of iodine, bromine and fluorine on the system of Lavoisier; but in all this, I see nothing but an extension of that system: nothing which Lavoisier, were he now alive, might not incorporate with his Elements, after having made a few trivial alterations. And while Dr. Ure indulges a morbid fancy,* about "the easy credulity with which the fictions of the Lavoisierian system have been received and propagated," I am of opinion, with Professor Silliman, that the views of the French philosopher, "remain substantially correct, and include all the cases (of combustion,) that mankind in general are acquainted with; although it is necessary to give extension to the idea of combustion, so as to embrace the action at least of chlorine, and perhaps, to some extent, of iodine and bromine."

Is there not reason to suppose, that the present state of things may be of ephemeral duration? If the general principles, and well known facts of the science, warranted the Lavoisierian chemists in the conjecture, that the earths and alkalies were metallic, have we not equal cause, at this moment, for doubting, in respect to the alleged elementary nature of chlorine, bromine, fluorine and iodine? I think it would require more than ordinary credulity, to inspire a negative reply. With great propriety has Dr. Paris cautioned medical men against the introduction into prescriptions, of the terms chloride, proto-chloride and bi-chloride, because of the great probability, that chlorine may yet prove to be a compound.* Discovery in our science, is only in its infantile state, and we hesitate not to advance the opinion, that in less than a century, the list of simple substances will undergo a retrograde movement, and finally fall to the numerical standard of the ancient philosophers.

---

*See Dictionary of Chemistry, 1831.

†See his Pharmacologia.

In giving a full treatise on Chemistry, it is fair to state the whole truth, where facts are in our possession. And, in writing such a work, we should be as little disposed to notice the facts in Lavoisier's Elements, respecting the metallic nature of the earths and alkalies, and to be silent in regard to the labors of Sir Humphrey Davy, as to pursue the fashionable course which we have ventured to condemn. And if it shall ever fall to the lot of the reviewer, to add another to the large catalogue of Chemical books already before the public, he pledges himself to do justice to the Lavoisierian school,in the matter now complained of, while it will be his pleasure to give the just meed of praise to every deserving candidate for fame.

In his second volume, our author evinces the character of the student, to very great advantage. His copious and highly interesting notes and references, are proofs of devotion to the object before him. And as a whole, the volume may be pronounced one of the very best chemical works in the English language. It brings the science down to the time of its publication, in its most perfect and complete form. The portion devoted to vegetable chemistry is peculiarly interesting, containing not only all the facts that could be gleaned from the French and English journals, but also the valuable experiments and results of our American *pharmaciens*, who are putting forth their energies in this branch of the science, to great advantage. The attentive reader of this second volume, cannot fail to discover the happy results of the application of chemistry to the business of the Pharmaceutist. The establishment of the College of Apothecaries, in Philadelphia, gave a new impulse to the art of compounding and manufacturing remedial agents, and has furnished the world with the results of the labours of Staples, Carpenter, Smith, and others, with a sure pledge of future researches, that may bear honorable comparison with the exploits of Pelletier himself. Ought not these facts to stimulate our Western Apothecaries to a noble emulation? Is it not high time that a College of Pharmacy was instituted in Cincinnati, and in all the large and flourishing towns of the *Great Valley?*

On the subject of mineral poisons, Professor Silliman is full and satisfactory, and we would hope that the time is not distant, when our medical men will acquaint themselves with the science of Toxicology, to such an extent, as to escape the sneer of the advocate in our courts of justice. This can be effected only by acquiring a just view of the principles of chemistry, and this should be regarded as decidedly fundamental in medical education, as the study of the bones and tissues of the human body.

We cannot conclude our very brief and hasty remarks on this excellent work, without an earnest recommendation to every medical man, and student of medicine, to purchase it, if he can conveniently do so. It presents an immense body of invaluable chemical and scientific matter, to which reference may be had with great profit, even though innovations should augment in number and importance, as the science moves onward, in the rapid march of discovery. There are some slight inaccuracies and defects, which the author will not fail to correct in another edition; and as we find the gold so greatly to overbalance the dross, we are free to express our cordial approbation, well aware of the acknowledged truth, that it is easier to find fault with a good book, than to write one which may fairly aspire to mediocrity.

